# WON-OCDMA System Based on MW-ZCC Codes for Applications in Optical Wireless Sensor Networks

**DOI:** 10.3390/s21020539

**Published:** 2021-01-13

**Authors:** Saleh Seyedzadeh, Andrew Agapiou, Majid Moghaddasi, Milan Dado, Ivan Glesk

**Affiliations:** 1 Faculty of Engineering, University of Strathclyde, Glasgow G1 1XQ, UK; saleh.seyedzadeh@strath.ac.uk (S.S.); andrew.agapiou@strath.ac.uk (A.A.); 2 TBA Group, 2623 AP Delft, The Netherlands; majid.moghaddasi@tba.group; 3 Faculty of Electrical Engineering and Information Technology, University of Žilina, 010 26 Žilina, Slovakia; milan.dado@uniza.sk

**Keywords:** optical sensors, vibration sensing, quality of service differentiation, wireless optical networks, free space optics, multiwavelength laser, optical code division multiple access (OCDMA)

## Abstract

The growing demand for extensive and reliable structural health monitoring resulted in the development of advanced optical sensing systems (OSS) that in conjunction with wireless optical networks (WON) are capable of extending the reach of optical sensing to places where fibre provision is not feasible. To support this effort, the paper proposes a new type of a variable weight code called multiweight zero cross-correlation (MW-ZCC) code for its application in wireless optical networks based optical code division multiple access (WON-OCDMA). The code provides improved quality of service (QoS) and better support for simultaneous transmission of video surveillance, comms and sensor data by reducing the impact of multiple access interference (MAI). The MW-ZCC code’s power of two code-weight properties provide enhanced support for the needed service differentiation provisioning. The performance of this novel code has been studied by simulations. This investigation revealed that for a minimum allowable bit error rate of $10^{-3}$, $10^{-9}$ and $10^{-12}$ when supporting triple-play services (sensing, datacomms and video surveillance, respectively), the proposed WON-OCDMA using MW-ZCC codes could support up to 32 simultaneous services over transmission distances up to 32 km in the presence of moderate atmospheric turbulence.

## 1. Introduction

Optical sensors have found their application in structural health monitoring (SHM) thanks to their small size, high accuracy and immunity to electromagnetic noise [1]. The use of optical multiplexing techniques has advanced the capacity, capabilities and performance of sensor networks in terms of the number of sensing points while lowering system complexity and cost [2]. Optical fibres that carry sensor signals have also been used as sensing elements themselves. The successful application of optical sensing in the construction industry has been reported for different monitoring purposes including gas leakage [3], temperature [4], strain [5], structure vibration [6], reinforced concrete beams [7] and building cladding systems [8].

It is essential to monitor civil structures to appraise the structural condition to be able to predict the internal damages at an early stage [9,10]. Several fibre-optic methods and tools have been developed for vibration monitoring. In 2001, a fibre Bragg gratings (FBG) coupled with a broadband light source were exploited to detect vibrations based on the light intensity modulation produced by vibrating FBG [11]. The feasibility of a fully distributed vibration sensing was evaluated based on a fibre diversity detection sensor [12]. An intensity-modulated fibre-optic accelerometer was also developed for vibration monitoring of wind turbine blades [13].

Collecting and transporting data from optical sensors for their processing is the role of the optical sensor network (OSN). Optical multiplexing techniques including time and wavelength division multiplexing [14] and optical code division multiple access (OCDMA) have been adopted to connect distributed sensing points [15]. In wavelength division multiplexing, each sensor is assigned a single wavelength from the source optical spectrum [16]. Time division multiplexing dedicates different time slots to individual sensors. For OCDMA sensor networks both, synchronous and asynchronous architectures have been proposed [17] with focus on exploring different coding techniques. For example, a spectral amplitude coding (SAC) offers a reasonable multiple access interference (MAI) cancellation, simplicity of implementation [18] and the ability to support a differentiated quality of service (QoS) by varying code-weight [19]. Using the variable-weight SAC (VW-SAC) coding an efficient OCDMA communication system for supporting the vibration sensing of unequally distributed points has been proposed [6].

Most research has focused on equally distributed sensor points, where the received optical power (ROP) by each sensor should be similar. However, in SHM, most structures are distributed with different distances, and therefore it is not possible to set up an equidistant sensor network. This leads to a near-far problem. It should be noted that the use of optical amplifiers to compensate for losses is possible but increases the system noise as well as the cost of the network. To resolve this problem and support the vibration sensing of unequally distributed points, an SAC-OCDMA system was developed and proposed [20]. Similarly, for the VW-SAC system, different code families [18,19,21,22] and detection techniques [23] have been proposed. A successful experimental proof of concept for its use in optical communication has been already reported [24].

Optical fibres have a large bandwidth. This provides a temptation to extend the already existing fibre infrastructure to support SHM. However, it might not be the most cost-effective to install optical fibre for monitoring hard-to-reach places. Wireless optical networks (WON) are an elegant solution where the implementation of fibre is impractical or not cost-effective [25]. The integration of OCDMA and WON systems can provide the desired solution [26,27]. Wireless OCDMA systems have already been deployed to support healthcare monitoring applications [28]. However, the majority of developed OCDMA codes have too high cross-correlation thus not suitable for WON and SHM applications. Inspired by ZCC [29] and MDW [30], this paper proposes a Multi-Weight-Zero Cross-Correlation (MW-ZCC) code for use in WON-OCDMA systems that is capable of carrying simultaneously SHM, including video surveillance data.

The paper is organised as follows. Section 2 describes the MW-ZCC code construction, Section 3 the architecture of WON-OCDMA for a sensor system and explains the code detection and design. Section 4 focuses on the main results, and Section 5 presents analytical results; this is followed by Section 6 and Section 7.

## 2. Code Construction

The novel MW-ZCC code for SAC-OCDMA system is matrix-based, and its construction follows a series of simple steps described below.

**Step 1:** The code is based on an X×N matrix where a number of lines *N* represents a number of codes (i.e., users or sensors ⋯) and a number of columns *N* represents a number of chips, respectively. Thus the base matrix produces *X* code sequences with code length *N*. The cornerstone of each matrix is a matrix, $H_{0}$ where:

(1) $$H_{0}=\left[\begin{array}{ccc} 0 & 1 & 1\\ 1 & 0 & 0 \end{array}\right]$$

This matrix is utilised for increasing the number of users and weights using a simple replacement technique.

**Step 2:** A new matrix $H_{j}$ can be generated from the existing $H_{\left(j-1\right)}$ matrix using a replacement technique as follows:

(2) $$H_{j}=\left[\begin{array}{ccc} 0 & H_{\left(j-1\right)} & H_{\left(j-1\right)}\\ H_{\left(j-1\right)} & 0 & 0 \end{array}\right]$$

**Step 3:** When the replacement ($H_{\left(j-1\right)}$) is done, there will be blank spaces in the newly generated matrix $H_{j}$ which are filled in by a series of ‘0’s according to the matrix size; therefore, all the created matrices are symmetrical.

For example, in the earlier substitution, R=1, we have:

(3) $$\begin{array}{cc} H_{j} & =\left[\begin{array}{ccc} 0 & H_{0} & H_{0}\\ H_{0} & 0 & 0 \end{array}\right]\\ \; & =\left[\begin{array}{ccccccccc} 0 & 0 & 0 & 0 & 1 & 1 & 0 & 1 & 1\\ 0 & 0 & 0 & 1 & 0 & 0 & 1 & 0 & 0\\ 0 & 1 & 1 & 0 & 0 & 0 & 0 & 0 & 0\\ 1 & 0 & 0 & 0 & 0 & 0 & 0 & 0 & 0 \end{array}\right] \end{array}$$

**Step 4:** To generate higher code weights and users, another replacement is required. Now we use R=2:

(4) $$\begin{array}{cc} H_{j} & =\left[\begin{array}{ccc} 0 & H_{1} & H_{1}\\ H_{1} & 0 & 0 \end{array}\right]\\ \; & =\left[\begin{array}{ccccccccc} 0 & 0 & 0 & 0 & H_{0} & H_{0} & 0 & H_{0} & H_{0}\\ 0 & 0 & 0 & H_{0} & 0 & 0 & H_{0} & 0 & 0\\ 0 & H_{0} & H_{0} & 0 & 0 & 0 & 0 & 0 & 0\\ H_{0} & 0 & 0 & 0 & 0 & 0 & 0 & 0 & 0 \end{array}\right] \end{array}$$

If *W* is the code weight, *X* is the number of codewords (i.e., the number of users), and *N* will be the length of the constructed code. The described code generation procedure produces multiple weight codes all with code-weights of power of 2:

(5) $$W_{i}=2^{i}\;0\leq i\leq R+1$$

The highest achievable code-weight, $W_{max}$ for the proposed code is, therefore, $2^{R}+1$. From this, the number of the required replacement, *R* can be calculated:

(6) $$R=Log_{2}W_{max}-1$$

Similarly, a number of the generated codes (signature), *X* (i.e., users) is obtained by $2^{R}+1$, and the code length can be calculated as $N=3^{R+1}$.

**Step 5:** To achieve code-weights increments without increasing the number of users, the matrix can simply be multiplied horizontally. For example, a double code weight and double code length can be achieved as

(7) $$W_{i}^{\prime }=2W_{i}$$

and

(8) $$N_{i}^{\prime }=2N$$

**Step 6:** If it is only desired to increase the number of users without the code-weight increase, the mapping technique is applied to double the number of users.

In Equation (9) matrix $H^{\prime }$ illustrates the increment of the code-weight while in Equation (10), matrix $\hat{H}$ denotes the increase in the number of users, as was explained in steps 5 and 6 respectively. Here, the the code-weight which were initially 4, 2 and 1 had been increased to 8, 4 and 2 while the number of users has been maintained as 4:

(9) $$\begin{array}{cc} \; & H^{\prime }=\\ \; & \left[\begin{array}{cccccccccccccccccc} 0 & 0 & 0 & 0 & 1 & 1 & 0 & 1 & 1 & 0 & 0 & 0 & 0 & 1 & 1 & 0 & 1 & 1\\ 0 & 0 & 0 & 1 & 0 & 0 & 1 & 0 & 0 & 0 & 0 & 0 & 1 & 0 & 0 & 1 & 0 & 0\\ 0 & 1 & 1 & 0 & 0 & 0 & 0 & 0 & 0 & 0 & 1 & 1 & 0 & 0 & 0 & 0 & 0 & 0\\ 1 & 0 & 0 & 0 & 0 & 0 & 0 & 0 & 0 & 1 & 0 & 0 & 0 & 0 & 0 & 0 & 0 & 0 \end{array}\right] \end{array}$$

(10) $$\begin{array}{cc} \; & \hat{H}=\\ \; & \left[\begin{array}{cccccccccccccccccc} 0 & 0 & 0 & 0 & 1 & 1 & 0 & 1 & 1 & \; & & \; & & \; & & \; & & \\ 0 & 0 & 0 & 1 & 0 & 0 & 1 & 0 & 0 & \; & & \; & & \; & & \; & & \\ 0 & 1 & 1 & 0 & 0 & 0 & 0 & 0 & 0 & \; & & \; & & \; & & \; & & \\ 1 & 0 & 0 & 0 & 0 & 0 & 0 & 0 & 0 & \; & & \; & & \; & & \; & & \\ & \; & & \; & & \; & & \; & & 0 & 0 & 0 & 0 & 1 & 1 & 0 & 1 & 1\\ & \; & & \; & & \; & & \; & & 0 & 0 & 0 & 1 & 0 & 0 & 1 & 0 & 0\\ & \; & & \; & & \; & & \; & & 0 & 1 & 1 & 0 & 0 & 0 & 0 & 0 & 0\\ & \; & & \; & & \; & & \; & & 1 & 0 & 0 & 0 & 0 & 0 & 0 & 0 & 0 \end{array}\right] \end{array}$$

All empty spaces are filled by “0”s. Thus, the number of users has increased from four to eight while the number of code-weight is maintained as four. The total length of the resulting code, $N_{T}$ after the mapping is dependent on the number of mapping, *M*, and can be attained by $N_{T}=M\times N$, where *N* is the length of the matrix before mapping. *M* can be attained by $X_{T}/W$. Here $X_{T}$ is the total number of users.

## 3. System Description

**Figure 1:**
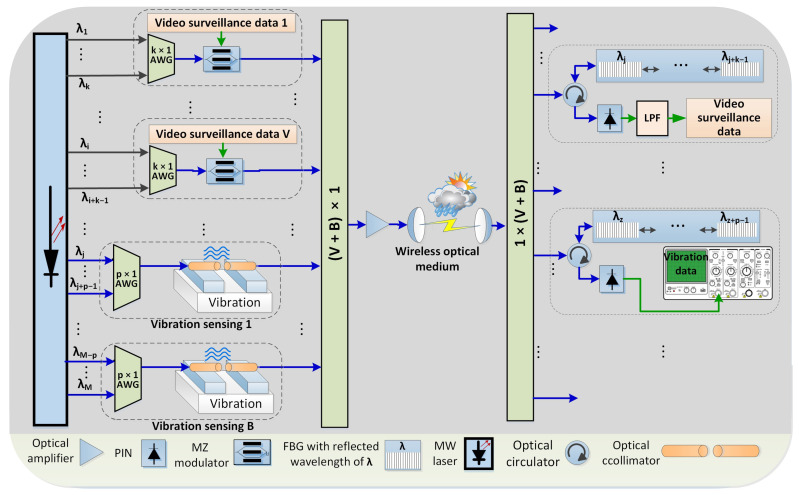
Diagram of WON-OCDMA hybrid system for video surveillance and vibration sensing monitoring.

Figure 1 illustrates the layout of the combined OCDMA and WON system. The OCDMA utilises a multiwavelength laser source based on [31]. This multiwavelength laser generates 90 wavelengths $\lambda_{1}$ to $\lambda_{M}$ spectrally separated by 20 GHz into so-called wavelength chips. Set of *k* wavelength is then distributed towards *V* video surveillance transmitters while a set of *p* wavelengths is distributed towards *B* transmitters used for structural vibration sensing. Note V×k+B×p=M were the number of generated wavelength by the laser.

Video surveillance data from each transmitter are carried by a dedicated MW-ZCC code with a code weight of *k*. Each code uses the assigned subset of wavelengths *i* to (i+k) from a dedicated k×1 array waveguide grating (AWG). A Mach-Zehnder modulator that follows performs NRZ on-off keying modulation of the Video surveillance signal on the code. Finally, signals from all video transmitters are combined by a (V+B)×1 power coupler and send for optical amplification before being broadcasted into free space towards the receiver. On a reception side, a 1×(V+B) power coupler sends a copy of the received spectrum to each decoder. The code detection is handled by fibre Bragg gratings (FBGs). Decoded data are sent to a user photodetector (PIN) for optical to electrical conversion. Finally, data are analysed. In this experiment, a bit error rate (BER) analyser is used to assess the quality of received signals in order to evaluate the system performance.

For vibration sensing, a set of *B* (p×1) AWGs is used to form a unique code with the code-weight of *p* at each vibration sensing point. (see Figure 1). There, a collimator mounted on the sensing box works as a code modulator to imprint the vibrations. In conducted simulations, a Mach-Zehnder modulator has been utilised to imitate the detrimental behaviour of the collimator used in vibration sensors. Three different frequencies of 70, 140 and 210 MHz were used to represent low, medium and high vibration, which are adopted from previous experimental research [6]. In the real world, the vibration intensity is not the discrete number; its range depends on the physical nature of the monitored structure and variations of the quiver. Before broadcasting, data from all vibration sensors are sent to an (V+B)×1 power coupler followed by an optical amplifier. On the receiving side, FBGs are used to filter out the individual codes. The decoded codes carrying vibration data are then converted into electrical domain by PIN photodetectors and sent for analyses; this experiment uses an oscilloscope with fast Fourier transform module.

## 4. Simulation Results

**Figure 2:**
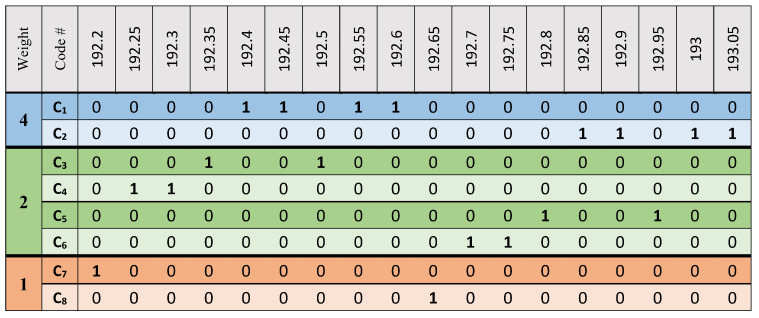
Codes composition for the proposed WON-OCDMA system for video surveillance, data and vibration services.

During the simulation, it was assumed there are four communication data channels coexisting with two video surveillance transmitters and two sensing points transmitters. Based on the MW-ZCC codes, code-weights of 4, 2, and 1 were assigned for video, data and sensing services, respectively. Used codes and associated wavelength code carriers are presented in Figure 2. The system performance was simulated using OptiSystem version 12 software, and default values were used for the optical and electrical components.

Three different vibration frequencies (see Table 1) monitored by two sensor nodes N1 and N2 were used to demonstrate the performance of the proposed system shown in Figure 1.

**Figure 3:**
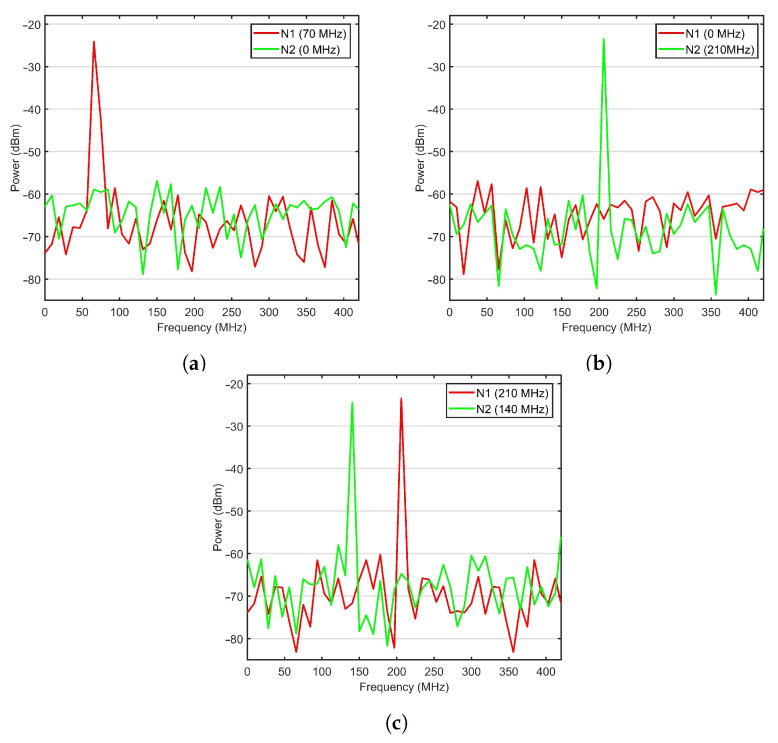
RF signal received from nodes N1 and N2 in experiments 1–3 (**a**–**c**) indicated in Table 1.

The bit rate for data and video services was set to 1.25 Gbps to follow the ANSI T1.105 standard of OC-24. The lasers power for each wavelength chip was set to 0 dBm. The bandwidth for AWGs at the vibration sensing points and FBGs at the monitoring side was considered as 50 GHz. The Mach-Zehnder modulator extinction ratio was 30 dB, and the noise figure for the optical amplifier was 4 dB. The WON transmitter and receiver aperture diameter were set to 5 and 20 cm, respectively. The PIN photodetectors were used with responsivity was 1 A/W and thermal noise of $10^{-22}$ W/Hz. In the WON channel, the assumed attenuation was set constant at 4 dB/km. The performance of the system was evaluated for WON affected by moderate turbulence (case when atmosphere index refraction structure is $10^{-14}$ m${}^{-2/3}$ [32]). The received power from sensing nodes N1 and N2 in Experiments 1 to 3 is shown in Figure 3a–c, respectively. The plotted RF signals are photodetector responses to received optical signals from two vibrations sensing nodes after 2 km transmission in WON under medium turbulence. Data represent weak, medium and high-level vibrations with frequencies of 68.1, 140.4 and 208.2 MHz, respectively. Figure 3a,b are related to scenarios in which only one sensor is affected by vibrations, while in Figure 3c both sensors are simultaneously affected (i.e., modulated). In all three scenarios, video data transmissions were active. By comparing those three plots, it can be seen that the signals from other sensors and video surveillance data have a negligible effect on the quality of retrieved signal for individual vibration sensors.

**Figure 4:**
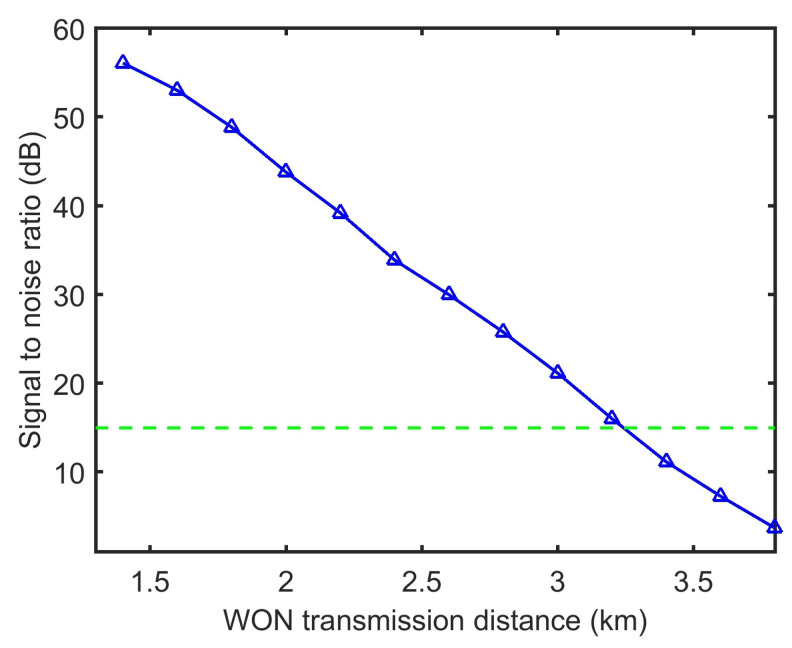
Example of SNR of the received signal from sensing none N2 as a function of transmission distance. The green line is the detectability limit.

Example of SNR of the signal s from a sensing node N2 under conditions depicted in Figure 3c received over WON as a function of transmission distance is shown in Figure 4. The green line is the detectability limit. Maintaining an SNR of 15 dB, which is considered as the minimum recoverable value [6].

**Figure 5:**
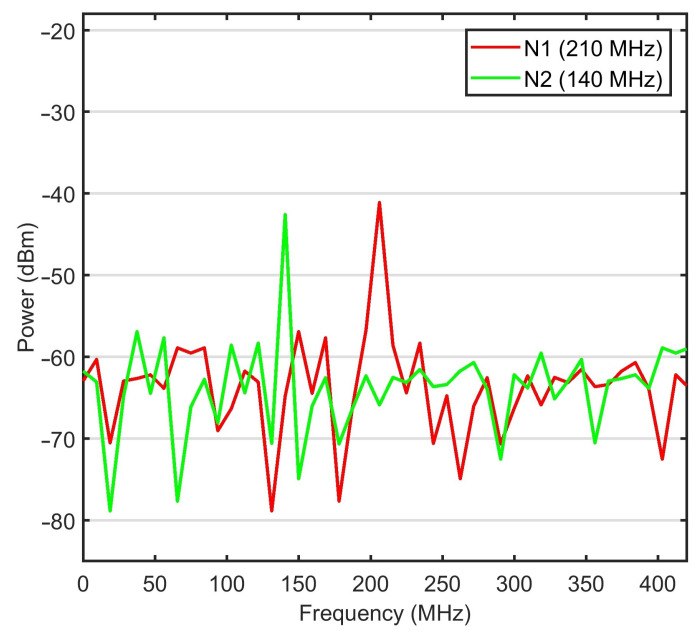
Example of received RF signal from sensing nodes N1 and N2 after 3.2 km transmitted in WON with moderate turbulence.

**Figure 6:**
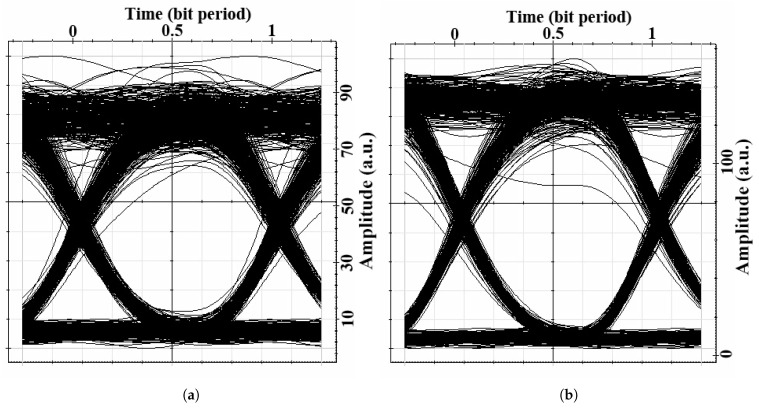
Eye diagram of signals related to (**a**) data service and (**b**) video surveillance data after 3.2 km propagation in atmosphere.

Example of received vibration signal (210 MHz and 140 MHz) from two sensing nodes N1 and N2, respectively after 3.2 km transmitted in WON with moderate turbulence is depicted in Figure 5. The eye diagrams for the worst-case scenario data and surveillance video are shown in Figure 6. After 3.2 km transmission, the BER values for data and video are $10^{-9}$ and $10^{-13}$, respectively. The signals from the vibration sensing were also retrievable at this distance with SNR of 15.4 and 16.1 dB for node N1 and N2, respectively.

## 5. Theoretical Analysis

To analyse the results obtained, we assume each sensing point acts as a data transmitter operating at the same data rate and using OOK modulation. This analysis aims to provide a lower bound of the system capacity.

The SNR of a consecutive fibre optic communication system is attained as [23]:

(11) $$SNR=\frac{{\langle I\rangle }^{2}}{\langle I^{2}\rangle }=\frac{I_{b}^{2}}{i_{nb}^{2}}$$

where $i_{nb}^{2}$ is the power of noise sources that exist in the photocurrent and $I_{b}$ is the mean acquired photocurrent at the receiver. It can be determined as:

(12) $$I_{b}=RbW_{h}P_{r}$$

where $P_{r}$ is the achieved optical power per wavelength, *R* is the photodiode responsivity, *b* is a bit value and is equal to 1, and where the specified subscriber transmit bit “1”, elsewhere it is zero. $W_{h}$ is the count of wavelengths for the users in group *h* which are retrieved by the photodetector. Their values depend on the detection technique and the coding technique. For complementary subtraction $W_{h}=W$ because all frequencies of the users are received by the photodetector. Nevertheless, in the direct decoding, that is the technique used for decoding the MW-ZCC, the $W_{h}$ figure is reliant on the used coding technique. For example, $W_{h}$ is equal to 1 when the utilised code has a modified quadratic congruence, 2 for MDW code and *W* for ZCC.

In addition, parameter $i_{nb}^{2}$ represents the variance of the total noise power, and for the SAC-OCDMA set-up which employs a multiwavelength laser, it is attained by [33,34]:

(13) $$i_{nb}^{2}=i_{RINb}^{2}+i_{shb}^{2}+i_{th}^{2}$$

where $i_{RINb}^{2}$, $i_{th}^{2}$ and $i_{shb}^{2}$ are the variance of the noises incidental to the relative intensity noise (RIN), thermal noise and shot noise. $i_{shb}^{2}$ is expressed as:

(14) $$i_{shb}^{2}=2EbW_{h}P_{r}B_{e}$$

$B_{e}$ denotes the electrical bandwidth, where *E* represents the electron’s charge. With the assumption that the number of disturbing signal transmitting bit “1” at every wavelength for all the users are the same, the mean value for *x* is:

(15) $$x=\frac{W_{h}^{2}\left(K-1\right)}{2N}$$

Since RIN occurs at the transmitter and all other interfering users cause crosstalk with the desired user signal, it is concluded that $W_{h}=W$ and

(16) $$i_{RINb}^{2}=RIN{\left(bWP_{r}+xP_{c}\right)}^{2}B_{e}$$

where RIN is the noise factor. The thermal noise can be achieved by:

(17) $$i_{th}^{2}=\frac{4K_{B}TB_{e}}{R_{L}}$$

where *T* is temperature, $K_{B}$ is the Boltzmann constant, and $R_{L}$ is the load resistor. Then the power variance of the total noise is calculated as:

(18) $$i_{n}^{2}=i_{RIN}^{2}+i_{sh}^{2}+i_{th}^{2}$$

To obtain the SNR, considering that the probability of sending bit ‘1’ by every user is 1/2, then the RIN, as well as the shot noise, must be divided by 2. Therefore, the total SNR can be achieved by:

(19) $$SNR=\frac{R^{2}W_{h}^{2}P^{2}{|}_{r}}{\frac{RIN}{2}{\left(WP_{r}\right)}^{2}B_{e}+EW_{h}P_{r}B_{e}+\frac{4K_{B}TB_{e}}{R_{L}}}$$

where $P_{r}$ in a SAC-CDMA operating in WON is obtained as:

(20) $$P_{r}=\frac{P_{t}D^{2}e^{-\Omega L}}{N{\left(\theta L\right)}^{2}}$$

Here, $P_{t}$ is the total sent power into the free space medium, *D* is the diameter of the utilised receiver aperture, *N* denotes the code length, Ω represents the attenuation coefficient, *L* is the total length of WON medium, and the θ is the beam divergence. To achieve BER as the system performance parameter, the air turbulence effect should be taken into account. The intensity of the turbulences is measured by a Rytov variance, which is attained by:

(21) $$\sigma_{R}^{2}=1.23C_{n}^{2}K_{own}^{7/6}L^{11/6}$$

here $C_{n}^{2}$ is the refractive index structure coefficient and $K_{own}=2\pi /\lambda$ is the optical wave number.

The system behaviour and reliability are denoted by the probability density function (PDF) of the randomly fading irradiance. Utilising Gamma−Gamma distribution, the pdf of the terms of normalised irradiance *I* is given by [35]

(22) $$P_{G}\left(I\right)=\frac{2{\left(\alpha \beta \right)}^{\frac{\left(\alpha +\beta \right)}{2}}}{\Gamma (\alpha )\Gamma (\beta )}I^{\left[\frac{\left(\alpha +\beta \right)}{2}-1\right]}K_{\alpha -\beta }\left(2\sqrt{\alpha \beta I}\right)$$

where Γ(.) is the Gamma function, and $K_{\left(\alpha -\beta \right)}$ is the qualified Bessel function of the second kind of order α−β. The α and β factors represent the effective number of small and large-scale twists of the scattering atmosphere and are calculated as

(23) $$\alpha ={\left\{exp\left[\frac{0.49\sigma^{2}}{{\left(1+1.11\sigma_{R}^{\frac{12}{5}}\right)}^{\frac{7}{6}}}\right]-1\right\}}^{-1}$$

and

(24) $$\beta ={\left\{exp\left[\frac{0.51\sigma^{2}}{{\left(1+0.69\sigma_{R}^{\frac{12}{5}}\right)}^{\frac{5}{6}}}\right]-1\right\}}^{-1}$$

The BER of an optical signal which has been transmitted in the Gamma˘Gamma distributed medium can be obtained as [36]:

(25) $$BER=\int_{0}^{\infty }BER_{0}\left(I\right)P_{G}\left(I\right)dI$$

where $BER_{0}\left(I\right)$ represents the BER of the signal conveyed in the additive white Gaussian noise (AWGN) channel and is calculated as

(26) $$BER_{0}\left(I\right)=Q\left(\sqrt{\frac{I^{2}\hat{\gamma }}{2}}\right)$$

where

(27) $$Q\left(\gamma \right)=\frac{1}{2}erfc\left(\frac{y}{\sqrt{2}}\right)$$

Here, $\hat{\gamma }$ is the average electrical SNR and erfc(y) is the complementary error function.

### Obtained Results

The values for the exploited parameters in the investigation were selected to allow for comparison with previous research and are summarised in Table 2.

**Figure 7:**
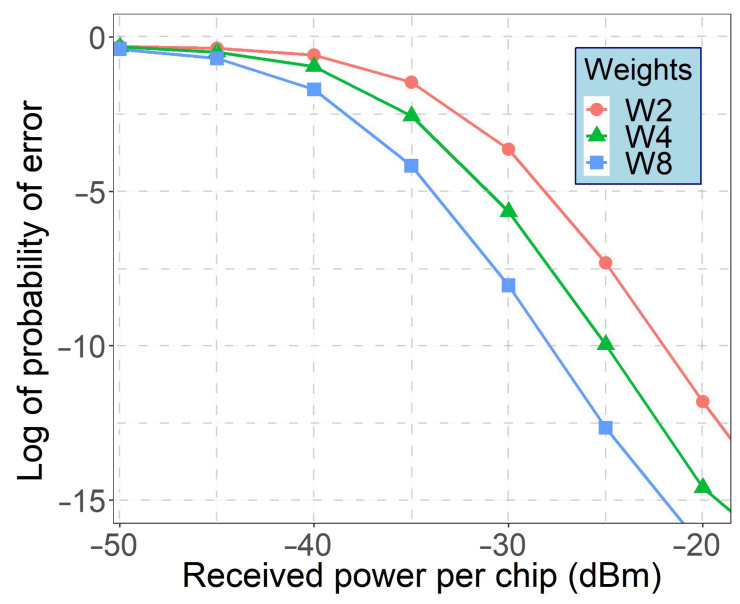
Probability of error versus ROP per code for concurrently transmitting users using code weight W of 2, 4 and 8.

Figure 7 illustrates the performance of WON-OCDMA system with 8 MW-ZCC code words as a function of the received optical power (ROP) per chip. As can be observed, users with higher code-weights provide greater performance.

**Figure 8:**
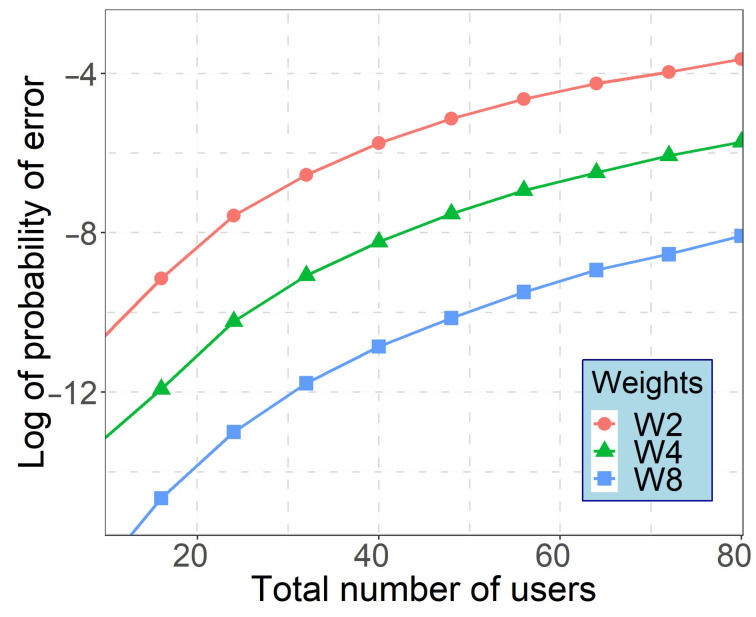
Probability of error versus total number of users as a function of code weight W of 2, 4, 8.

The calculated number of simultaneous users (data services and vibration point sensors) using MW-ZCC codes as data carriers is shown in Figure 8. It was assumed that services are evenly represented in the systems. Assuming the minimum allowable BER of $10^{-3}$, $10^{-9}$ and $10^{-12}$ to support triple-play services, WON-OCDMA employing novel MW-ZCC codes can support up to 32 simultaneous subscribers. In the OOK modulation, a BER of $10^{-3}$ is equivalent to an SNR of 15 dB.

**Figure 9:**
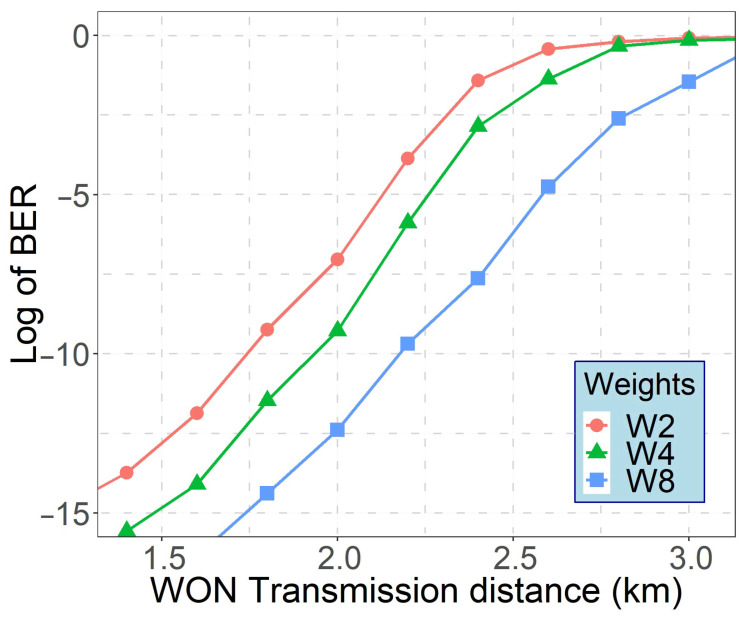
Probability of error versus transmission distance in moderate turbulence for concurrently transmitting users with weights 2, 4 and 8.

The performance degradation due to increased transmission distance in the atmosphere under moderate turbulence with 32 simultaneous users’ (including transmission from 10 sensing points) was investigated using simulation set-up in OptiSystem software. The result is illustrated in Figure 9, indicating a successful transmission up to 2 km.

## 6. Discussion

The research presented in this study addresses the functionality gap between emerging optical sensor networks and urban SHM in order to leverage efficient remote vibration sensing of constructed sites. Despite the broad adoption of optical sensing in SHM, there are several challenges when it comes to monitoring various structures located in hard-to-reach areas. To address the needs, this paper proposes a new family of MW-ZCC codes for use in advanced monitoring systems for carrying information collected by optical sensors monitoring structural vibration. The proposed codes were investigated in WON-OCDMA system that does not require any traffic management or synchronisation.

It was shown that the proposed approach could also be effectively implemented without any undesirable interference or crass talk on other simultaneous communication services, including remote video surveillance. The novel family of MW-ZCC codes can support high-performance optical transmission systems that need to provide QoS differentiation in free space applications. As shown, MW-ZCC codes are well suited for use in WON-OCDMA systems even when used as single-weight codes because of their low cross-correlation properties. Since they are easy to convert into codes with the power of two code-weigh, they are well suited to support the desired QoS differentiation.

## 7. Conclusions

This paper proposes a novel MW-ZCC coding scheme with a low cross-correlation function for WON-OCDMA system. Codes are easy to convert into multiweight power of two codes, thus suitable for supporting a variety of QoS services in WON, including sensing, datacomms and video surveillance applications.

The effect of a free space transmission with medium turbulence on the signal transmission and received optical power was analysed. The simulations results revealed that for a minimum allowable BER of $10^{-3}$, $10^{-9}$, when supporting triple-play services (sensing, datacomms and video surveillance), the proposed WON-OCDMA employing MW-ZCC codes could carry up to 32 services simultaneously at a distance of 32 km in the presence of moderate turbulence in the atmosphere.

The research results presented in this paper thus offer conceptual approaches for engineering of future OCDMA-based wireless optical networks supporting triple-play services.

## Figures and Tables

**Table 1:** Vibration frequencies monitored by nodes N1 and N2.

Node	Exp1	Exp2	Exp3
N1	70 MHz	0 MHz	210 MHz
N2	0 MHz	210 MHz	140 MHz

**Table 2:** Summary of the parameters used for system investigation.

Symbol	Parameter	Value
θ	Beam divergence	0.5 mrad
λ	Operating wavelength	1550 nm
*D*	Receiver aperture diameter	8 cm
$B_{e}$	Electrical bandwidth	2.5 GHz
$\sigma_{R}^{2}$	Rytov variance	1
Ω	Weather attenuation	3 dB/km
$P_{c}$	Crosstalk power	−30 dBm
$P_{t}$	Total transmitted power	20 dBm

## Data Availability

Not applicable.
